# Elevated butyric acid and histamine in feces and serum as an indicator of onset of necrotic enteritis in broiler chickens

**DOI:** 10.3389/fmicb.2025.1581309

**Published:** 2025-05-09

**Authors:** Hemlata Gautam, Shaik Noor Ahmad, Babajan Banaganapalli, Shelly Popowich, Betty Chow-Lockerbie, Lisanework E. Ayalew, Rupasri Mandal, David S. Wishart, Suresh Tikoo, Susantha Gomis

**Affiliations:** ^1^Department of Veterinary Pathology, Western College of Veterinary Medicine, University of Saskatchewan, Saskatoon, SK, Canada; ^2^Department of Genetic Medicine, Faculty of Medicine, King Abdulaziz University, Jeddah, Saudi Arabia; ^3^Department of Pathology and Microbiology, Atlantic Veterinary College, University of Prince Edward Island, Charlottetown, PE, Canada; ^4^Departments of Biological Sciences and Computing Science, University of Alberta, Edmonton, AB, Canada; ^5^Department of Vaccinology and Immunotherapy, School of Public Health, University of Saskatchewan, Saskatoon, SK, Canada

**Keywords:** broiler chickens, necrotic enteritis, metabolic pathways, butyric acid, histamine, feces, serum, disease diagnosis

## Abstract

**Background:**

*Clostridium perfringens* (CP) induced necrotic enteritis (NE) is an economically significant intestinal disease of broiler chickens. Identifying potential biological markers during the development of NE might facilitate early disease control measures. Therefore, the current study aimed to identify the metabolites and metabolic pathways changes associated with the onset of NE in serum and feces of CP-infected broiler chickens.

**Methodology:**

The protein content of the feed was abruptly altered from 20% to 28% using a well-established NE model before challenging the birds with CP. Then, we performed a targeted, fully quantitative liquid chromatography-tandem mass spectrometry (LC-MS/MS) -based assay for analyzing the metabolomics profile of serum, feces, and jejunal contents in NE birds. The data were analyzed to understand the trend of metabolite distribution, relationships between metabolites and pathway impacts.

**Results:**

Birds with NE showed metabolic variations including lipids, amino acids, and organic acids, across all the biological samples analyzed. This variation was higher in serum samples (310/597 metabolites, 51.92%), compared to fecal (182/608 metabolites, 29.93%), and jejunal samples (125/607 metabolites, 20.59%). A robust statistical analysis of these metabolites identified 19 common metabolites, including butyric acid and histamine. Pathway analysis identified that six of them were enriched in key pathways, like tricarboxylic acid cycle (TCA cycle) (citric acid and cis-aconitic acid), glyoxylate and dicarboxylate metabolism (citric acid and cis-aconitic acid), arginine-proline metabolism (spermine and creatinine), butanoate metabolism (butyric acid), and histidine metabolism (histamine). These pathways were related to energy synthesis, nitrogen metabolism, and immune response in NE birds.

**Conclusion:**

This study highlights metabolic differences in birds with NE and underscores butyric acid and histamine as potential early biomarkers for NE diagnosis. The upregulation of these metabolites across serum, jejunal and fecal samples reflects their local and systemic impacts on the disease. These biomarkers play key roles in several NE hallmark features, including gut barrier disruption, dysbiosis of microbes and tissue injury through immune system activation, and systemic inflammation. Future studies need to validate our findings across field conditions and different predisposing factors.

## 1 Introduction

Necrotic enteritis (NE) is a major economically important enteric disease of poultry that predominantly affects broiler chickens during the grow-out period. Estimated global production losses due to NE are approximately US$6 billion annually (McDevitt et al., 2006; Wade and Keyburn, 2015). Overproduction of tissue-degrading and pore-forming toxins by the pathogenic strains of *Clostridium perfringens* (CP) are responsible for pathology of the intestine during the disease progression (Popoff, 2014). It has been reported that 61% of healthy broiler chickens at 3–4 weeks of age carry CP isolates with *netB* while 95% of broiler chickens with NE are associated with CP with *netB* (Abildgaard et al., 2010; Chalmers et al., 2008). Hence, CP as an opportunistic pathogen needs predisposing factors that can either damage the physical/mucosal barrier of the gut or deplete the immune cells of the host or provide nutrition for it to multiply. Predisposing factors include coccidial infection and dietary changes (high protein diets) that can directly change the physical properties of the gut, either damaging the epithelial surface, enhancing mucus production, or changing gut transit times, disrupting the gut microbiota, and altering the immune status of birds (Moore, 2016).

NE is characterized by a sudden increase in mortality, poor weight gain, and overall production losses in broiler chickens that account for severe financial losses (Wade and Keyburn, 2015). Traditionally, presumptive diagnosis of NE is based on history, classical gross and histopathology, however, isolation of CP is required for the confirmation (Dufour-Zavala, 2008). Further, bacteriology from the intestine would also be uncertain due to the presence of CP as a normal part of the microbiome. For the disease diagnostic perspective of the poultry industry, easy and non-invasive methods are required for the timed prediction of NE. Due to the complex nature of NE, pathogenesis is poorly understood. In recent years, the metabolomics approach has shown versatility in finding small molecules to diagnose various cancers and gastrointestinal diseases in humans (D’Incà and Sturniolo, 2023; Wang et al., 2023).

Metabolites are the intermediate or end products of the metabolic processes and are connected to the functional status of a biological system (Qiu et al., 2023). In the context of disease pathogenesis, these small molecules are produced either at the initiation of the disease or during the progression. These metabolic reactions are the complex interaction of nutrients, the intestinal metabolism, and the microbiota composition that leads to specific metabolites in various biological fluids or feces that allow differentiation between health and disease (De Preter and Verbeke, 2013). In both animals and humans, blood is a primary biofluid to determine health status due to the presence of numerous molecules that are required for the maintenance of normal physiological functions. One recent study identified a total of 7,191 metabolites in the serum of the chickens (Tian et al., 2023). In a study, authors demonstrated that arachidonic acid metabolism is activated in co-infection of *Mycoplasma gallisepticum* and *Escherichia coli* in chickens. Furthermore, gene expression and arachidonic acid network analysis revealed a direct relationship with the leukotriene pathways and an inverse relationship with the infection. Hence, this study suggested leukotriene C4 in serum as a potential biomarker for detecting poultry respiratory diseases (Wu et al., 2020).

In addition, the serum metabolites comprise a wide variety of endogenous metabolites that are influenced by intrinsic and extrinsic factors. However, recent studies have shown that human serum metabolome is primarily determined by the human gut microbiome (Bar et al., 2020). In one study, a cohort of 1,569 healthy individuals was investigated for the association of microbiome and genome metabolite interactions in paired samples of blood and feces. The authors reported that 595 metabolites were significantly related to host genetics or gut microbiome, among which 69, 15, and 16% were contributed only by the microbiome, host genetics, and both, respectively (Diener et al., 2022). Similarly, in chickens, feeding of virginiamycin or plant essential oils not only increased the relative abundance of Bacteroidetes and decreased the abundance of Firmicutes in the cecum but also modified the cecal (bile acid synthesis) and serum (biosynthesis of unsaturated fatty acids) metabolites in comparison to the control birds (Chen et al., 2020). Another research supplemented young broilers’ diet with butyrate glycerides (3,000 parts per million) up to 20 days (d), shown to significantly increase the abundance of *Bifidobacterium* in the ileal microbiota. Further, nuclear magnetic resonance revealed a significant increase of alanine, low-density lipoproteins and lipids metabolites (Yang et al., 2018).

Fecal metabolomics provides a non-invasive approach to predict the enteric diseases in humans as well as in animals. Previously, it was documented that the potential of volatile organic compounds (VOCs) such as ethanoic, butanoic, pentanoic acids, acetone, or benzaldehyde as biomarkers in the human feces to determine the presence of bacterial disease such as ulcerative colitis, *Campylobacter jejuni*, and *C. difficile*. The authors further proposed to develop a rapid diagnostic device based on VOC detection in feces (Garner et al., 2007). Similar study investigated the VOCs in chicken feces to determine the presence of *C. jejuni* or *E. coli* of zoonotic importance. The study identified six VOCs including hexanal, (E)-2-octenal, pyrrole, ethyl ethanoate, methyl alcohol and 2-heptanone in *C. jejuni* or *C. coli* in compared to the negative farms or control birds (Garner et al., 2008). Media reports highlighted recent research identifying NE-specific VOCs that included reduced sulfur compounds in the feces/manure as well as in the environmental samples (Canadian Poultry Magazine, 2023). These above-mentioned studies aimed to find the various predictors in infectious poultry diseases while information is lacking in prospect of NE.

Previously, we documented the role of butyric acid in the jejunum of broiler chickens during the progression of NE (Gautam et al., 2025). In the current study, our objective is to explore the metabolic alterations in serum and feces as an advanced liquid chromatography-tandem mass spectrometry (LC-MS) based The Metabolomics Innovation Centre (TMIC) mega targeted metabolomics approach. Early detection of NE will benefit both poultry and human health by mitigating pathogenic proliferation, optimizing therapeutic efficacy, safeguarding production sustainability, and promoting food security and safety. Current diagnostic procedures regarding NE require pathological examination followed by bacterial culture and histopathology of infected tissues, which takes 48–72 h to obtain results. Biomarker-based diagnostics procedures can be conducted in real time. By identifying key metabolite changes and associated pathways, we aim to uncover potential biomarkers that could facilitate early and non-invasive detection of NE, ultimately improving health management and NE control in the broiler chicken industry.

## 2 Material and methods

**FIGURE 1:**
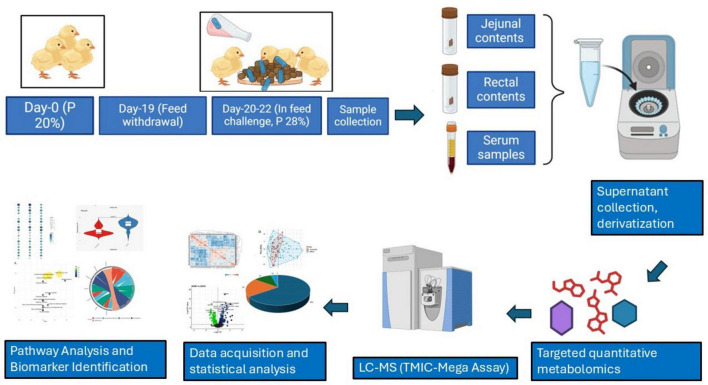
Workflow for metabolomics Analysis of different biological samples of NE Birds. The diagram highlights the timeline and dietary manipulation, sample collection (serum, jejunal, and rectal contents), sample processing (supernatant extraction and derivatization), targeted quantitative metabolomics using LC-MS (TMIC-Mega assay), statistical analysis, and pathway identification for biomarker discovery.

The overall workflow for this study is presented in Figure 1.

### 2.1 Animal care

All animal experiments conducted for this study were granted by the Animal Research Ethics Board at the University of Saskatchewan and Canadian Council on Animal Care guidelines followed. Animal work was conducted at the Animal Care Unit (ACU), Western College of Veterinary Medicine (WCVM), University of Saskatchewan. Birds are raised on soft wood shavings of depth 3–5 cm. From placement until 3 days of age, chicks received 23 h of light and 1 h of darkness at 40 lux. After 3 days of age, the darkness period increased to 8 h and the light intensity decreased to 30 lux during the 16 h light period. The initial temperature was set at 30–32°C for first 3 days and decreased by 0.5°C per day until a temperature of 21°C was maintained.

### 2.2 Animal model of CP in broiler chickens

**FIGURE 2:**
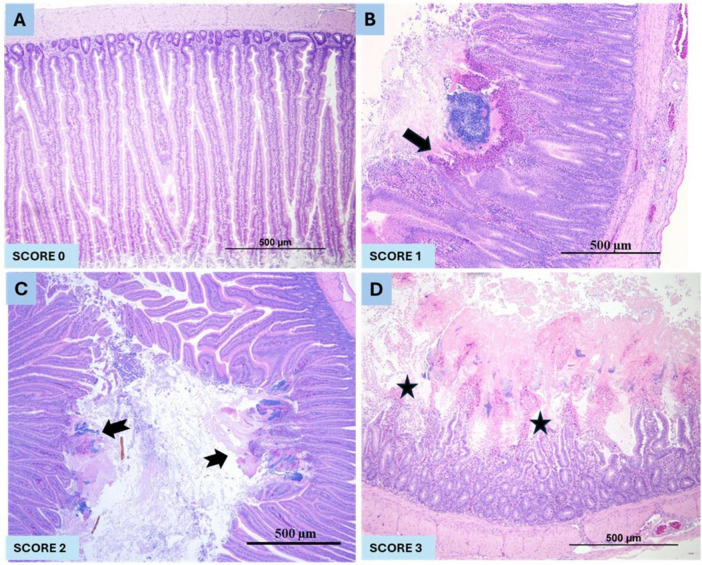
The histopathological NE lesions score in pre-CP and post-CP infection in broiler chickens. **(A)** represents the normal intestinal mucosa (Score 0), **(B)** showing mild, focal (arrow), acute necrosis of intestinal villi (Score 1), **(C)** showing moderate (notched arrow), multifocal to coalescing, moderate, acute necrosis of intestinal villi (Score 2) and **(D)**. representing severe, diffuse (stars), acute necrosis and loss of intestinal villi (Score 3).

A well-established animal model of NE in broiler chickens was used to study the metabolic profile and pathways for this study (Gautam et al., 2025). Briefly, broiler chickens were fed a commercial raised without antibiotics (RWA) broiler starter ration containing 20% protein (Farm Choice™ RWA, Masterfeeds, Canada) until 18 days. Feed was withdrawn at 19 days. At 20 days of age, a new feed ration was introduced containing 28% protein. The 28% RWA feed was prepared by mixing a commercially available 25% RWA turkey starter (MasterFeeds, Canada) with 38% poultry supplement (MasterFeeds, Canada) at a 10:3 ratio. In this model, a sudden increase of protein in the feed, from 20% to 28% immediately before the CP challenge, was used as a predisposing factor. In order to minimize variables, an abrupt increase in protein content was conducted in both control and CP challenge groups. A CP isolate (CP 21) containing *cpa*, *netB*, *cpb2*, and *tpeL* toxin genes was grown in fluid thioglycollate (FTG) media (Sigma-Aldrich, Oakville, ON, Canada). The culture was added to the ration (1:1 v/w) and fed twice daily for 3 consecutive days (20–22 days of age) as previously described (Gautam et al., 2025). Birds were observed for clinical signs and mortality three times per day until the termination of the experiment at 23 days of age. Mortality, gross and histopathological scoring of the intestine were conducted as previously described (Gautam et al., 2025). Briefly, histopathological intestinal lesions were scored as: 0 = no lesions/healthy mucosa; 1 = focal necrosis of intestinal villi, acute; 2 = necrosis of intestinal villi, multifocal to coalescing, acute; and 3 = diffuse, necrosis of intestinal villi, acute, severe in all birds (Figures 2A–D).

### 2.3 Experiment A

The objective of this experiment was to study the metabolic profile and pathways associated with NE development in commercial broiler chickens. On the day of hatch, a total of 47 broiler chicks were randomly divided into two groups: (1) no CP challenge (*n* = 23); (2) CP challenge (*n* = 24). At the termination of the experiment at 23 days of age, sections of intestines were collected for histopathology from all the birds. Microscopic lesions of NE were scored as described above. In order to study the metabolic derangements, the contents of the jejunum and rectum (fecal contents) of the broiler chickens were collected along with blood samples from individual birds (Figure 2C). To study the metabolic derangements, intestinal contents from jejunum, rectum/fecal samples and blood samples were collected from broiler chickens.

### 2.4 Experiment B

The objective of the second experiment was to investigate (1) the consistency and repeatability of the metabolites and metabolic pathways identified during the development of NE; (2) to increase the statistical power in bioinformatics analysis. On the day of hatch, a total of 60-broiler chicks were randomly assigned into two groups (*n* = 30/group) (1) no CP challenge; (2) CP challenge. At the end of the experiment at 23 days of age, sections of the jejunum were collected to confirm the NE by histopathology as described above in the experiment A. To study the metabolic profile, intestinal contents from jejunum, rectum/fecal samples along with the blood samples were collected from broiler chickens.

### 2.5 Metabolomics sampling and processing

At 23 days of age, three types of samples were collected from each bird for metabolomics analysis: (1) blood/serum samples, (2) rectal/fecal samples, and (3) jejunal samples. Before euthanasia, blood samples were collected from the brachial vein of each bird. Further, blood samples were centrifuged for 5 min, and serum was collected and then flash-frozen immediately using dry ice and ethanol. Also, rectum/fecal and jejunal samples were collected in 1 mL Eppendorf tubes and immediately placed on ice. Gross lesions of NE in the intestine were recorded. Intestinal sections were collected for histopathology to score NE lesions. Sections of jejunum are carefully removed, and intestinal contents are collected gently into sterile 5 mL eppendorf tubes and placed on ice. Rectal/fecal and jejunal contents were centrifuged for 5 min at 1,968 *g*. The flash frozen samples were stored in −80°C until shipped to TMIC, University of Alberta, Canada on dry ice for analysis.

### 2.6 Metabolomic analysis

The TMIC Mega Metabolomics assay is employed to identify and quantify up to 900 targeted endogenous metabolites using a combination of direct injection (DI) mass spectrometry and reverse-phase LC-tandem mass spectrometry (LC-MS/MS) (Applied Biosystems/MDS Analytical Technologies). This assay can detect metabolites encompassing a broad range of biochemical classes, including amino acids, derivatives, sugars, biogenic amines, organic acids, nucleobases, vitamins, cofactors, amine oxides, short-chain fatty acids, acylcarnitines, and various lipid types (sphingomyelins, triglycerides, glucosylceramides, ceramides, cholesterol esters, and diglycerides). A detailed description of the TMIC Mega Metabolomics assay is provided in a previous publication (Gautam et al., 2025).

In brief, isotope-labeled internal standards (ISTDs) with concentrations ranging from 1 to 10 μM and chemical derivatization reagents such as PITC for amino acids and 3-NPH for organic acids were added to enhance ionization and separation during mass spectrometry analysis. Stock solutions with concentrations from 0.01 to 1 mM for each analyte were prepared by dissolving accurately weighed chemicals in appropriate solvents. Seven calibration curve standards (Cal1 to Cal7, with concentrations ranging from 0.01 to 100 μM) and three quality control (QC) standards (low, medium, and high concentrations: 0.05, 0.5, and 5 μM, respectively) were prepared by mixing and diluting stock solutions with appropriate solvents. For amino acids, amino acid derivatives, biogenic amines, and nucleotide/nucleosides, PITC derivatization was performed by drying the samples under a nitrogen stream, adding a 5% PITC derivatization solution, and extracting the targeted analytes with methanol containing 5 mM ammonium acetate. LC-MS/MS analysis was then conducted by transferring 50 μL of extracts to a new 96-deep-well plate and diluting with 450 μL of LC/MS-grade water to quantify these metabolites. For organic acids, 3-NPH derivatization was utilized by adding a derivatization reagent consisting of 250 mM 3-NPH in 50% aqueous methanol, shaking the mixture at room temperature for two hours, adding LC/MS water and 2 mg/mL butylated hydroxytoluene (BHT) dissolved in methanol, and further diluting the mixture. DI-MS/MS analysis was conducted by transferring 10 μL of the remaining extracts to another 96-deep-well plate and diluting with 490 μL of direct flow injection (DFI) buffer to quantify lipids, acylcarnitine’s, and glucose/hexose.

To ensure accuracy and precision, three QC samples at low, medium, and high concentrations were included, and each sample was analyzed in triplicate for reproducibility. Internal standards were used to normalize the data, and limits of detection (LOD) and quantification (LOQ) were determined for each metabolite. Blank samples were included to monitor potential contamination and carryover effects. The robustness and reproducibility of the method were validated through these rigorous quality control measures, as outlined in previous publications (De Preter and Verbeke, 2013).

### 2.7 Metabolomics data analysis

Metabolomic data, quantifying metabolite concentrations in micromolar (μM) units, underwent rigorous preprocessing to ensure data reliability and comparability. To address missing values below the LOD, imputation with half the minimum positive value was employed for each metabolite. To stabilize variance and normalize data distribution, logarithmic transformation was applied.

Differential metabolite analysis was conducted using the limma package in R to identify metabolites significantly altered between serum and tissue groups (Ritchie et al., 2015). To account for multiple comparisons, *p*-values were adjusted using the false discovery rate (FDR) method, with a significance threshold of FDR < 0.05. To visualize overall metabolic profiles and identify potential outliers, unsupervised principal component analysis (PCA) was performed.^1^ For pairwise group comparisons, volcano plots were generated to highlight significantly altered metabolites based on fold change and adjusted *p*-values were employed to display expression patterns across different sample types for individual samples.

Subsequently, differential metabolite analysis was conducted using the limma package in R to identify metabolites that were significantly altered between serum and tissues. Univariate analysis was performed to assess the distribution, normality, and homogeneity of variance of each metabolite. To mitigate the risk of false positives due to multiple comparisons, the FDR method was applied, setting a significance threshold of FDR < 0.05. To visualize overall metabolic trends and identify potential outliers, unsupervised PCA was performed. To identify metabolites with significant differential abundance between experimental groups, pairwise volcano plots were generated. These plots display both the magnitude of fold change and statistical significance (adjusted *p*-value) for each metabolite.

To further explore metabolite relationships and patterns, heatmaps were created using MetaboAnalyst to visualize the top metabolites, and correlation matrices were generated to assess the strength of associations among metabolites. Additionally, regression analysis was conducted to investigate the relationship between metabolite levels and relevant outcomes. To compare metabolite levels between control and NE infected groups, violin plots accompanied by Wilcoxon test (two-group comparison, non-parametric) were utilized employed to compare levels of metabolites of interest between control and NE groups.

To gain biological insights into the identified differentially expressed metabolites, pathway enrichment analysis was performed using MetaboAnalyst with *Gallus gallus* as the reference organism. Significantly enriched pathways were determined based on a *p*-value cutoff of < 0.05. Visualizations such as GO chord plots were generated using Cytoscape.

All the statistical analyses and visualizations are primarily performed using R statistical software, with the aid of packages including limma (Ritchie et al., 2015), ggplot2 (Wickham, 2016), enhanced volcano,^1^ heatmap (Gu et al., 2016), pathway analysis and network visualization were conducted using XCMS (Smith et al., 2006), MetaboAnalyst (Chong et al., 2018) and Cytoscape (Shannon et al., 2003), respectively.

## 3 Results

### 3.1 Experiment A

There was no mortality in group 1 birds (no CP challenge), in contrast, group 2 birds (CP challenge) had 20% mortality (CP challenge). No gross or microscopic NE lesions were observed in group 1 (no CP challenge). In contrast, 100% of birds in group 2 (CP challenge) exhibited NE lesions, with 85% showing a score of 3 and 15% a score of 2.

### 3.2 Experiment B

There was no mortality in group 1 (no CP challenge) in contrast, group 2 (CP challenge) had 20% mortality. No gross or microscopic NE lesions in group 1 (no CP challenge) in contrast, 100% of birds in group 2 (CP challenge) had NE lesions (score 3 in 90% birds and score 2 lesions in 10% birds) (*p* < 0.0001) (Figures 2A–D).

### 3.3 Metabolomics analysis of samples of NE and control birds

#### 3.3.1 Serum samples

**FIGURE 3:**
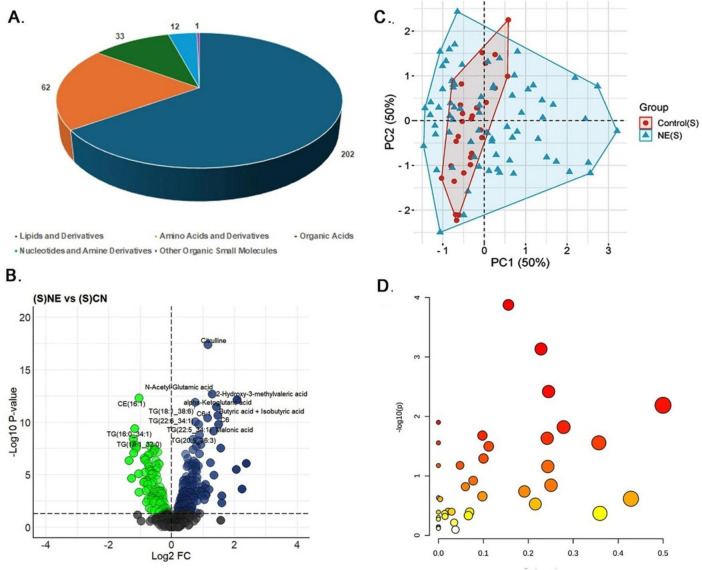
Metabolomics analysis of serum samples of NE birds. **(A)** Pie chart illustrating the distribution of 310 differentially expressed metabolites identified in NE bird serum samples. These metabolites include lipids and derivatives (202 metabolites, 65.16%), amino acids and derivatives (62 metabolites, 20%), organic acids (33 metabolites, 10.65%), nucleotides and amine derivatives (12 metabolites, 3.87%), and other organic small molecules (1 metabolite, 0.32%). **(B)** Volcano plot showing 196 upregulated and 114 downregulated metabolites in NE birds versus control serum samples. **(C)** PCA plot showing multivariate analysis of serum metabolite data. Although there is partial overlap between NE and control samples, NE samples demonstrated greater metabolic variability. Principal components 1 and 2 (PC1 and PC2, respectively) together explain 50% of the total variance, highlighting clear metabolic differences between NE and control groups. **(D)** Bubble plot representing the pathway impact scores and *p*-values of disrupted metabolic pathways in serum samples of NE birds.

Serum samples underwent variance and abundance filtering to enhance the quality of metabolomics data between NE and control groups (Supplementary Figure 1). Univariate analysis identified significant alterations in 310 out of 597 metabolites (51.92%) in NE birds compared to controls (*P* < 0.05) (Supplementary Table 1A). These 310 included 202 lipids and derivatives (65.16%), 62 amino acids and derivatives (20%), 33 organic acids (10.65%), 12 nucleotides and amine derivatives (3.87%), and 1 small molecule (0.32%) (Figure 3A). Of these, 196 metabolites (63.22%) were upregulated, with a log fold change (LogFC) range of 0.07 to 2.38, while 114 metabolites (36.77%) were downregulated, with a LogFC range of −1.35 to −0.09 (Figure 3B). The heatmap illustrates the expression patterns of the top 20 differentially expressed metabolites in NE-affected serum samples (Supplementary Figure 2).

Multivariate analysis using PCA showed that while there is some overlap between NE-affected and control samples, NE samples exhibited greater metabolic variability. Both Principal Component (PC) 1 and PC2 accounted for 50% of the variance in the serum metabolite data, indicating distinct metabolic differences between the groups (Figure 3C). Correlation analysis further identified significant associations among specific metabolites in serum (Supplementary Table 1B). For example, among the triglycerides, TG (16:0–34:1) and TG (18:2–34:2) exhibited a very high positive correlation (*r* = 0.98), suggesting their co-regulation in lipid metabolic processes. Similarly, isoleucine and leucine demonstrated a strong positive correlation (*r* = 0.98), highlighting their linked roles in protein synthesis and energy production, which are critical responses to NE-induced stress. Additionally, alpha-ketoglutarate and 2-oxoisocaproate showed a strong positive correlation (*r* = 0.98), indicating their interconnected roles in the TCA cycle and amino acid metabolism. For histamine, the top positively correlated metabolites were creatine (*r* ≈ 0.7) and spermine (*r* = 0.6), indicating coordinated changes in energy storage and cellular metabolism, while norepinephrine (*r* ≈ −0.52) exhibited the strongest negative correlation. In the case of butyric acid and isobutyric acid, N-acetyl-glutamic acid (*r* = 0.75) and 2-hydroxy-3-methylvaleric acid (*r* = 0.7) showed strong positive correlations, whereas TG (16:0–34:1) (*r* = −0.56) was the most negatively correlated (Supplementary Figure 3).

Regression analysis reinforced these findings, with citrulline exhibiting a strong positive correlation with NE, as evidenced by a regression coefficient of 2.28 and a highly significant *p*-value of 3.43E-06. Other metabolites, such as TG (20:2–34:1) and LysoPC a C20:3, also showed significant associations, implicating their roles in altered lipid metabolism and membrane signaling (Supplementary Table 1C). Finally, pathway enrichment analysis revealed significant disruptions in arginine and proline metabolism, glycine, serine, and threonine metabolism, the citrate cycle, and butanoate metabolism, suggesting alterations in protein synthesis, amino acid metabolism, energy production, and other essential processes in serum samples of NE birds (*P* ≤ 0.05) (Figure 3D and Supplementary Table 1D).

#### 3.3.2 Jejunal samples

**FIGURE 4:**
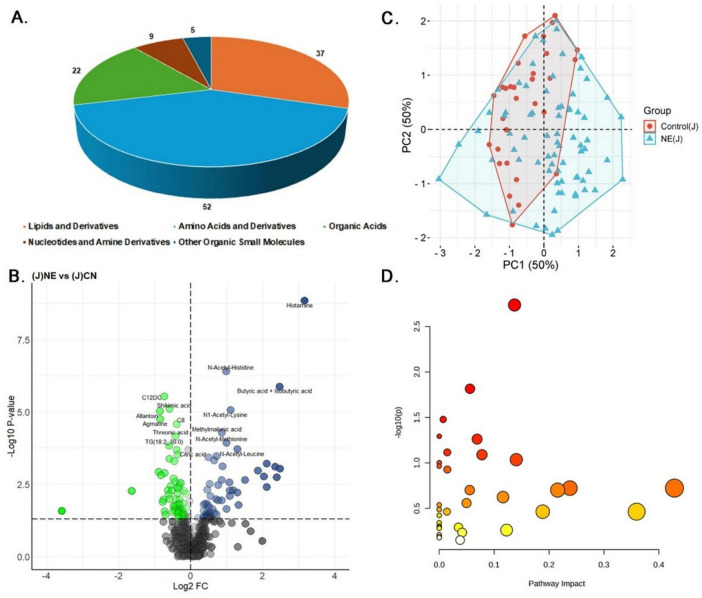
Metabolomics analysis of jejunal samples in NE Birds. **(A)** Pie chart showing the distribution of 125 significantly altered metabolites identified in NE affected jejunal samples, including lipids and derivatives (52 metabolites, 44.44%), amino acids and derivatives (37 metabolites, 31.62%), organic acids (22 metabolites, 18.80%), nucleotides and amine derivatives (9 metabolites, 7.69%), and other organic small molecules (5 metabolites, 4.27%). **(B)** Volcano plot displaying 58 metabolites (55.56%) significantly upregulated and 67 metabolites (48.44%) significantly downregulated in NE birds compared to control jejunal samples. **(C)** PCA plot illustrating multivariate analysis of jejunal metabolite data. Although some overlap exists, NE samples exhibit greater metabolic variability. Principal components 1 and 2 (PC1 and PC2, respectively) together explain 50% of the variance, differentiating NE affected birds from control groups based on their metabolic profiles. **(D)** The Bubble plot represents the pathway impact scores and *p*-values of disrupted metabolic pathways in jejunal samples of NE birds.

In the jejunal samples, data normalization was done to ensure the reliability and consistency of the metabolomics data, thereby improving the comparability between NE-affected and control groups (Supplementary Figure 4). Univariate analysis identified significant alterations in 125 out of 607 metabolites (20.59%) in NE-affected jejunal contents (Supplementary Table 2A). The distribution of these 125 differentially expressed metabolites included 52 lipids and derivatives (44.44%), 37 amino acids and derivatives (31.62%), 22 organic acids (18.80%), 9 nucleotides and amine derivatives (7.69%), and 5 other organic small molecules (4.27%) (Figure 4A). Of these, 58 metabolites (55.56%) were up-regulated, with a LogFC range of 0.19 to 3.16, while 67 metabolites (48.44%) were downregulated, with a LogFC range of −0.33 to −3.57 (Figure 4B). The heatmap depicts the expression patterns of the top 20 differentially expressed metabolites, showcasing distinct upregulation and downregulation patterns between NE-affected and control jejunal tissues (Supplementary Figure 5).

PCA of the jejunal content data showed a substantial overlap between NE-affected and control samples. However, NE samples tended to cluster more toward the positive side of PC1, indicating some degree of metabolic differentiation. The considerable overlap along PC2, however, suggested that the metabolic profiles of NE-affected tissues shared similarities with those of controls (Figure 4C). Correlation analysis further highlighted strong associations among N-acetylated amino acids in the jejunal contents (Supplementary Table 2B and Supplementary Figure 6). The strongest correlation was observed between N-acetyl-proline and N-acetyl-glycine (*r* = 0.97), suggesting a combined role in collagen synthesis and cellular detoxification. Additionally, N-acetyl-tyrosine and N-acetyl-proline exhibited a notable correlation (*r* = 0.97), pointing toward their involvement in protein synthesis and stress modulation. Histamine displayed a positive correlation with N-acetyl-methionine (*r* = 0.92), while choline showed a negative correlation (*r* = −0.49). Butyric acid and iso-butyric acid demonstrated a positive correlation with alpha-amino-isobutyric Acid (*r* = 0.81) and a negative correlation with quinoline-4 carboxylic acid (*r* = −0.55). Regression analysis further underscored the importance of histamine in NE, with a regression coefficient of 0.1165 and a significant *p*-value of 0.003 (Supplementary Table 2C). Propionic acid and betaine also demonstrated a strong positive association, hinting at their role in the NE-affected metabolic pathways. Finally, jejunal pathway enrichment analysis has shown considerable disruptions in arginine and proline metabolism, beta-alanine metabolism, and glutathione metabolism (*p*-values ≤ 0.05). These findings suggest disruptions in protein synthesis, energy metabolism, and oxidative stress response, all of which are critical in the pathology of NE (Figure 4D) (Supplementary Table 2D).

#### 3.3.3 Fecal samples

**FIGURE 5:**
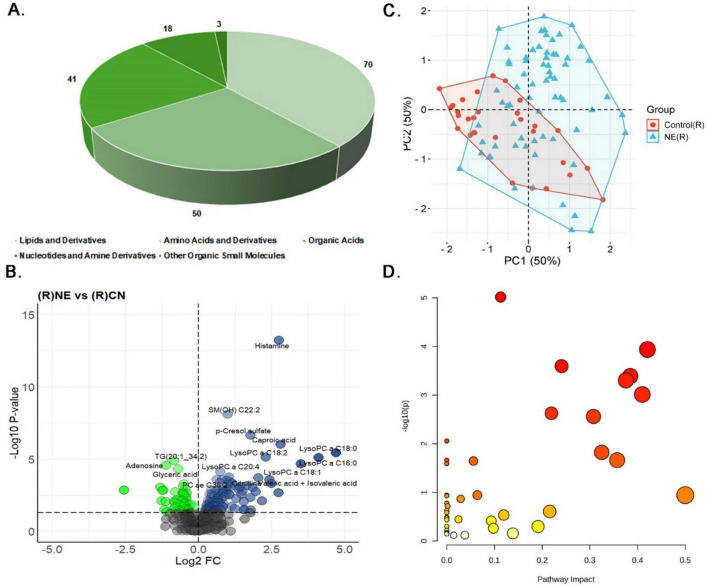
Metabolomics analysis of fecal samples of NE Bird. **(A)** Distribution of differentially expressed metabolites: lipids and derivatives (70, 44.87%), amino acids and derivatives (50, 32.05%), organic acids (18, 11.54%), nucleotides and amine derivatives (3, 1.92%), and other organic small molecules (41, 6.75%). **(B)** Volcano plot displaying 122 metabolites (67.03%) significantly upregulated and 60 metabolites (32.96%) significantly downregulated in NE birds compared to control fecal samples. **(C)** PCA plot of fecal metabolite profiles. Principal Component 1 (PC1) partially separates NE-affected from control samples; Principal Component 2 (PC2) shows considerable overlap, reflecting shared metabolic features. **(D)** The Bubble plot represents the pathway impact scores and *p*-values of disrupted metabolic pathways in fecal samples of NE birds.

Fecal samples underwent variance and abundance filtering to improve data quality (Supplementary Figure 7). Univariate analysis revealed significant alterations in 182 out of 608 metabolites (29.93%), including 70 lipids and derivatives (44.87%), 50 amino acids and derivatives (32.05%), 18 organic acids (11.54%), 3 nucleotides and amine derivatives (1.92%), and 41 other small organic molecules (6.75%) (Figure 5A and Supplementary Table 3A). In total, 122 metabolites (67.03%) were upregulated, with LogFC values ranging from 0.05 to 4.70, while 60 metabolites (32.96%) were downregulated, with LogFC values ranging from −2.54 to −0.11 (Figure 5B). The heatmap of the top 20 differentially expressed metabolites illustrates the expression patterns between NE-affected and control fecal samples (Supplementary Figure 8). PCA analysis showed some separation between NE-affected and control samples, with a lot of overlaps, indicating similar metabolic features (Figure 5C).

Correlation analysis of fecal samples showed many strong links between important metabolites (Supplementary Table 3B and Supplementary Figure 9). For example, there was a very strong positive link (*r* = 0.98) between leucine and isoleucine, indicating coordinated regulation in response to NE. Similarly, PC as C34:2 and PC as C36:2 showed a strong positive correlation (*R* = 0.95), as did LysoPC a C16:0 and LysoPC a C18:1 (*r* = 0.94), suggesting interconnected roles in metabolic pathways. Conversely, asparagine exhibited strong negative correlations with Cer (d18:1/24:1) (*r* = −0.68), and TG (16:1–34:3) had strong negative correlations with PC aa C32:1 (*r* = −0.68) and PC aa C36:1 (*r* = −0.69). Additionally, TG (16:0–37:3) demonstrated a strong negative correlation with PC ae C34:1 (*r* = −0.68), and TG (20:3–32:2) exhibited the strongest negative correlation (*r* = −0.65). N-Acetyl putrescine showed the strongest positive correlation with butyric acid and iso-butyric acid (*r* = 0.70), while C6:1 had a strong positive correlation with histamine (*r* = 0.65). Regression analysis further supported these findings, identifying histamine as a key player in the inflammatory processes of NE, with a regression coefficient of 1.9110 and a highly significant *p*-value of 0.0002. Orotic acid (coefficient: 0.48, *P* = 0.01) and SM(OH) C22:2 (coefficient: 1.14, *P* ≤ 0.02) also demonstrated significant associations, implicating disruptions in nucleotide synthesis and membrane integrity (Supplementary Table 3C). Finally, pathway enrichment analysis in fecal samples highlighted significant disruptions in several metabolic pathways, including glutathione metabolism, alanine, aspartate, and glutamate metabolism, glyoxylate and dicarboxylate metabolism, and arginine biosynthesis (*p* ≤ 0.05). These disruptions may collectively affect detoxification, amino acid metabolism, energy production, and protein synthesis, providing further insights into the metabolic alterations occurring in fecal samples in NE birds (Figure 5D and Supplementary Table 3D).

### 3.4 Comprehensive analysis of differentially expressed metabolites across serum, jejunal contents and fecal samples in NE birds

**FIGURE 6:**
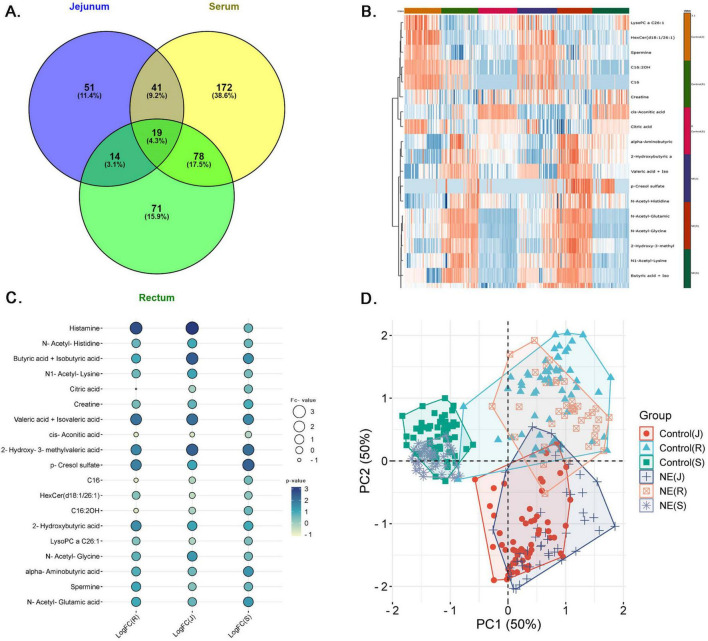
Comprehensive analysis of common metabolites across serum, jejunal contents and feces in NE birds. **(A)** Venn diagram shows the distribution of differentially expressed metabolites across jejunum, feces, and serum, with 19 common metabolites. **(B)** Heatmap displays clustering of upregulated (red) and downregulated (blue) metabolites across NE and control samples. **(C)** Dot plot highlighting 19 key metabolites, including histamine and butyric acid, across jejunal, serum, and fecal samples. Bubble size corresponds to expression ratios, and color intensity indicates the LogFC values. **(D)** PCA plot illustrating clear metabolic separation between NE birds and control chickens across jejunal, fecal, and serum samples. PC1 and PC2 (each 50%) explain major variance, with distinct NE clusters (circles, crosses, stars) separate from controls (squares, triangles, rectangles). Tight clustering of controls indicates minimal variation, while dispersed NE clusters reflect significant disease-induced metabolic changes.

We further investigated the significant metabolites across serum, jejunal contents and fecal samples of the rectum in NE birds to find the common metabolites. The Venn diagram in Figure 6A illustrates the distribution of significant metabolites across serum, rectum, and jejunal samples in NE birds. Serum had the highest number of unique metabolites, with 172 metabolites (38.6%), followed by fecal samples from the rectum with 71 metabolites (15.9%), and jejunal contents with 51 metabolites (11.4%). Particularly, 19 metabolites (4.3%) were found common among all the 3 sample types, suggesting them as universal biomarkers for NE. Moreover, 41 (9.2%) metabolites were common between serum and jejunal contents, 14 (3.1%) metabolites between rectal and jejunal contents, and 78 (17.5%) metabolites between serum and feces. This distinct metabolite distribution highlights both localized and systemic metabolic influences of the NE disease. The heatmap illustrates distinct metabolite expression pattern between NE and control samples, confirming the NE-induced metabolic shifts in chicken (Figure 6B).

The results illustrated in Figure 6C show differential expressions of 19 metabolites in serum, fecal, and fecal contents of chickens with NE (Supplementary Table 4A). The bubble plot shows the relationship expression levels of selected metabolites, where bubble size corresponds to the expression ratios and color intensity, indicating the degree of up and down-regulation. This analysis points to the metabolites, which are upregulated among all samples, which position them as potential NE biomarkers. Of these metabolites, histamine is seen to be the most prominent upregulated metabolite with LogFC of 3.16 in Jejunum, 2.72 in the rectum, and 0.69 in serum, all with high significance *p*-values (1.43E-09, 6.01E-14, and 0.0013). Similarly, butyric acid and iso-butyric acid have also shown up-regulation (LogFC: 2.46 in the jejunal contents, 0.96 in the feces, and 1.49 in serum), with respective *p*-values like 1.32E-06, 0.0027, and 2.37E-11. Similarly, valeric acid and isovaleric acid have also shown this metabolic trend (LogFC: 2.10 in the jejunal contents, 2.05 in the feces, and 1.60 in serum) with significant *p*-values (0.0006, 0.0002, and 0.0010). The consistent upregulation of these metabolites across the jejunal, feces, and serum samples not only underscore their importance in disease pathogenesis but also confirms them as potential NE biomarkers.

The PCA plot in Figure 6D shows clear metabolic separation across the jejunal, feces, and serum samples between NE and control group chicken. PC1 and PC2 each explain 50% of the variance, capturing the major differences in the dataset. The jejunal contents, feces, and serum samples (circles, crosses, stars) in the NE cluster are distinct from control samples (squares, triangles, rectangles) in all samples, indicating significant metabolic shifts due to NE. The tight clustering of control samples suggests minimal variation in healthy chickens, while the more dispersed NE clusters reflect the disease’s impact. This separation confirms that NE causes significant and systemic metabolic changes.

The correlation analysis across the jejunal contents, feces, and serum of NE-affected chickens reveal notable patterns, with both commonalities and distinct relationships across these tissue fluids as shown in Supplementary Figure 10. In the Jejunum, the primary site affected in NE, strong correlations between key metabolites, such as HexCer (d18:1/26:1) and LysoPC a C26:1 (*r* = 0.88), creatine and spermine (*r* = 0.91), indicate a tightly regulated response in lipid metabolism and energy/cellular repair processes (Supplementary Figure 10A). In the serum, the correlation between HexCer (d18:1/26:1) and LysoPC a C26:1 decreases to *r* = 0.78, indicating systemic but less intense metabolism regulation, while the correlation between creatine and spermine remains moderate (*r* = 0.71). The negative correlation between butyric acid and iso and N-acetyl-histidine is weaker in serum (*r* = −0.20664), suggesting that the disruptions in energy metabolism are more localized to the jejunal contents and feces. Histamine shows strong correlations in the serum with creatine (*r* = 0.65) and spermine (*r* = 0.61), reflecting its role in systemic immune regulation and energy metabolism (Supplementary Figure 10B). Negative correlations, such as butyric acid and iso and N-acetyl-histidine (*r* = −0.46) and 2-hydroxy-3-methyl and spermine (*r* = −0.54), further highlight disruptions in energy metabolism and immune response. Notably, histamine shows a moderate positive correlation with spermine (*r* = 0.45) in the jejunum, linking immune activation with tissue repair. In feces, similar lipid metabolism patterns are observed, with HexCer (d18:1/26:1) and LysoPC a C26:1 showing a nearly identical correlation (*r* = 0.88), though creatine and spermine are less strongly correlated (*r* = 0.44), and the impact on 2-hydroxy-3-methyl and spermine is minimal (*r* = −0.05). Histamine also shows a weaker correlation with spermine (*r* = 0.23), suggesting a less pronounced immune-metabolic interaction in feces (Supplementary Figure 10C). Overall, although NE affects systemic metabolic changes, the significant disruptions take place in jejunal contents, with diminishing activity in fecal and serum samples.

### 3.5 Pathway impact analysis of 19 metabolites common across serum, feces, and jejunal contents reveal key metabolic disruptions in NE-affected birds

**FIGURE 7:**
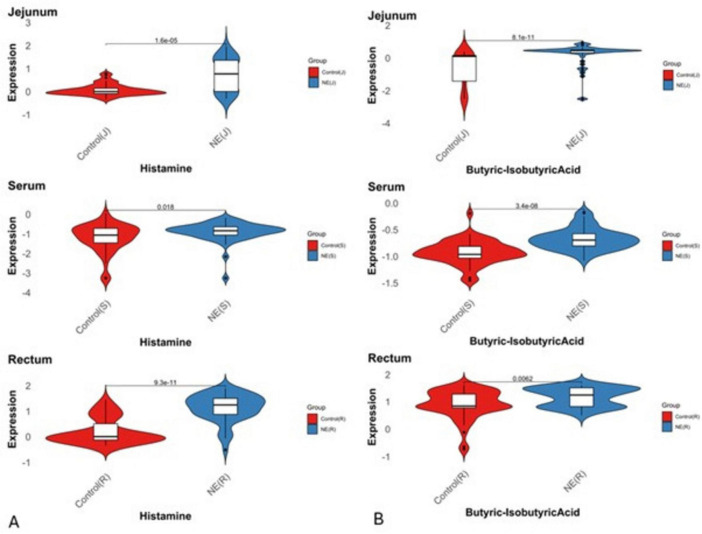
Expression status of histamine and butyric acid metabolites across serum, fecal and jejunal samples in NE birds. Violin plots illustrate the distribution and central tendency differences in fold changes (log2 scale) of **(A)** histamine and **(B)** butyric acid across serum, fecal, and jejunal samples between NE and control groups (*P* < 0.05). Positive values on the *y*-axis indicate upregulation, while negative values represent downregulation.

**FIGURE 8:**
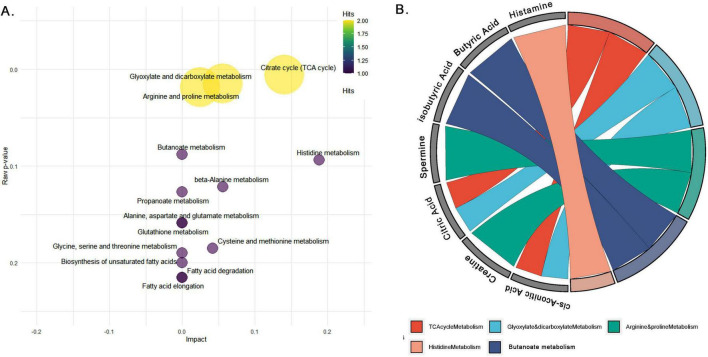
Pathway analysis of common metabolites across serum, jejunal contents, and feces in ne-affected birds. **(A)** Pathway impact analysis of differentially expressed metabolites common to serum, feces and jejunal contents, showing pathway impact (*x*-axis) and significance (*y*-axis). Circle size and color represent the number of metabolite hits per pathway, emphasizing critical disruptions in the TCA cycle, glyoxylate and dicarboxylate metabolism, arginine and proline metabolism, histidine metabolism, and butanoate metabolism. **(B)** GO Chord diagram linking key metabolites to their respective metabolic pathways, illustrating the involvement of citrate, butyric acid, histamine, spermidine, and spermine in pathways like the citrate cycle, arginine-proline metabolism, butanoate metabolism, and histidine metabolism.

The pathway impact analysis of 19 common metabolites across serum, jejunal contents, and feces in NE-affected birds highlights significant metabolic disruptions, with 6 metabolites (31.6%) enriched in key pathways including the TCA cycle (citrate cycle), glyoxylate and dicarboxylate metabolism, arginine and proline metabolism, histidine metabolism, and butanoate metabolism. Their expression levels of these metabolites across the serum, jejunum and fecal samples of NE and control birds are shown in Figures 7A, B and Supplementary Figure 11. These pathways point to critical disruptions in energy production, nitrogen metabolism, fatty acid utilization, and immune regulation. The pathway enrichment scatter plot (Figure 8A) visually demonstrates the significance and impact of these pathways, with the TCA cycle and histidine metabolism emerging as highly impacted pathways (Supplementary Table 4B). The TCA cycle had two hits (citric acid and cis-aconitic acid), with a pathway impact score of 0.14 and a highly significant *p*-value of 0.005, indicating substantial alterations in energy production and oxidative phosphorylation. Glyoxylate and dicarboxylate metabolism, which involved citric acid and butyric acid, had an impact score of 0.05 and a *p*-value of 0.01, suggesting disruptions in gluconeogenesis.

In the arginine and proline metabolism pathway, spermine and creatinine is involved, showing an impact score of 0.02 and a *p*-value of 0.01, reflecting disruptions in polyamine synthesis and nitrogen metabolism, critical for cellular repair processes. Butanoate metabolism featured butyric acid, which had a *p*-value of 0.08 but no significant pathway impact, indicating changes in gut energy metabolism and fatty acid utilization. Histamine, involved in the histidine metabolism pathway, demonstrated a relatively high pathway impact of 0.18, underscores its involvement in immune regulation and inflammatory responses, both of which are crucial in the pathophysiology of NE.

The gene ontology (GO) chord plots (Figure 8B) provide further visualization of the metabolite-pathway correlations, illustrating how specific metabolites (e.g., histamine, citrate, spermine) are connected to the key metabolic pathways like TCA cycle, histidine metabolism, and arginine and proline metabolism. These relationships emphasize the significant metabolic disruptions occurring across these pathways in NE-affected birds. The remaining 13 metabolites (68.4%), including N-acetyl-histidine, N1-acetyl-lysine, valeric acid and isovaleric acid, 2-hydroxy-3-methylvaleric acid, *p*-cresol sulfate, C16, HexCer (d18:1/26:1), C16 2OH, 2-hydroxybutyric acid, LysoPC a C26:1, N-acetyl-glycine, N-alpha-aminobutyric acid, and N-acetyl-glutamic acid, were not mapped to any KEGG pathways, indicating a lack of identified metabolic roles in the current pathway databases.

## 4 Discussion

One of the strategic priorities of the broiler chicken industry is enhancing disease control by managing pathogens at the onset of the disease to reduce economic losses, improve profitability of the sector, and improve food safety. The early detection of pathogenic infections is critical in controlling pathogens and, thus, imminent disease outbreaks and antimicrobial use. The chicken industry is currently relying on serological blood testing to measure antibodies against pathogens to detect pathogenic infection. However, serological tests detect diseases only 10–14 days after pathogenic exposure. Besides, PCR (pathogen DNA detection) and bacterial culture-based diagnosis methods are primarily contingent on the types of tissue and the pathogen’s predilection site. The broiler chicken industry cannot detect pathogens within 1–2 days of post-infection. The metabolomics approach will provide novel rapid disease diagnostic tools to detect microbial infections before the onset of clinical signs to implement disease control strategies and provide tools to monitor poultry performance, which will improve competitiveness, food safety, and profitability of the broiler chicken industry.

Our objective was to explore the serum and fecal metabolome, and the metabolic pathways associated with pathological lesions of NE in the jejunum following CP challenge containing toxin genes cpa, netB, cpb2, and tpeL. This approach was designed to help elucidate whether the metabolomic landscape of NE assists with the diagnosis of NE early in the onset of development of NE. Recently, we have demonstrated a strong correlation of elevated butyric acid in the jejunum of broiler chickens with NE following CP challenge irrespective of toxin gene combinations of CP (Gautam et al., 2025). Furthermore, in this NE animal model, some birds had no microscopic or macroscopic NE lesions, some had only microscopic NE lesions, and some birds had both macroscopic and microscopic NE lesions following CP challenge. We found that fold changes of butyric acid increased according to severity of NE lesions, lowest levels of butyric acid in birds with no microscopic or macroscopic NE lesions, then birds with only microscopic NE lesions, then the highest levels of butyric acid in birds with both macroscopic and microscopic NE lesions. Although butyric acid content increased according to the severity of NE, butyric acid content was significantly higher in all three variables compared to the control group with no exposure to CP [19]. Based on our metabolomics data, it is clear that butyric acid and histamine increased in the jejunum, serum, and feces in birds that developed NE irrespective of the severity of NE. Moreover, these findings were consistently replicated across two independent experiments, highlighting the reliability and robustness of our results.

CP has a wide variety of virulence factors including extracellular toxins such as pore forming or membrane damaging toxins. Also, CP produces enzymes including collagenases, hyaluronidases, adhesion molecules, quorum sensing molecules, iron acquisition systems, and regulatory proteins (*VirR/VirS*) that influence pathogenicity (Camargo et al., 2024). Studies support the role of these factors in toxin production and host responses. Understanding these virulence factors and their impact on host metabolism is crucial for developing effective NE control strategies and development of diagnostic tools to detect onset of NE in acute stages of the disease. In the present study, metabolic variability evident across the across serum, jejunal and fecal samples of NE birds reflect the systemic and localized impacts of CP infection. The pathway analysis of metabolites common among all these samples identified butyric acid (butanoate metabolism), histamine (histidine metabolism), citric acid and cis-aconitic acid (TCA cycle and glyoxylate and dicarboxylate metabolism), spermine and creatinine (arginine-proline metabolism) as potential candidates for NE. However, butyric acid and histamine are prioritized due to their specific roles in gut barrier disruption, microbial dysbiosis, tissue and immune system activation, and systemic inflammation key features of NE progression (Liu et al., 2024; Recharla et al., 2023). On the other hand, citric acid and cis-aconitic acid, though involved in metabolic pathways, are less directly implicated in NE pathogenesis (Sharifuzzaman et al., 2025). Spermine and creatinine, though important in cellular processes and in muscle metabolism, respectively, have little relevance to NE or intestinal inflammation. Furthermore, we have previously noted that both butyric acid and histamine were elevated in CP-challenged (with any combination of CP virulence genes) birds with no macroscopic or microscopic NE lesions.

Butyric acid (molecular weight, 88.11 g/mol; chemical formula, C4H8O2) is a short-chain fatty acid produced via microbial fermentation of dietary fibers by anaerobic bacteria most especially *Clostridium* spp., *Lachnospiraceae*, and *Ruminococcaceae* (Gautam et al., 2025; Huang et al., 2019). Butyrate improves intestinal health dynamics by increasing epithelial energy metabolism and upregulating claudin-1 and occludin, other tight junction proteins. However, the accumulation of butyrate during the NE may indicate microbial dysbiosis since infections of CP often lead to increased populations of butyrate-producing bacteria; hence, increased levels of butyrate result in excessive accumulation of SCFA (Camargo et al., 2024; Huang et al., 2019). Our pathway analysis shows that butyric acid is enriched in butanoate metabolism, which supports epithelial tissue repair in normal conditions but in CP-induced NE condition, its elevation may exacerbate intestinal damage by fueling mucin-degrading enzymes like *NanI* and *NanJ*, which promotes colonization and toxin production (Low et al., 2021; Yamaguchi and Yamamoto, 2023).

Elevated butyric acid levels increase intestinal permeability, allowing bacterial toxins into the bloodstream and thus setting off systemic inflammation (Gautam et al., 2025; Huang et al., 2019). Butyric acid exhibited the greatest elevation in the jejunum (logFC = 2.46), indicating localized metabolic disruption. Rectal levels (logFC = 0.96) were moderately elevated, supporting its use in non-invasive diagnostics, while serum (logFC = 1.49) reflects systemic metabolic shifts linked to NE. It also modulates inflammatory cytokines such as TNF-α and IL-1β through the NF-κB pathway. It is early elevation, even in CP-challenged birds without NE lesions, suggests potential as a preclinical biomarker (Camargo et al., 2024; Gautam et al., 2025). Moreover, butyric acid detection in fecal samples offers practical advantages over serum analysis, particularly for farm-level diagnostics of NE. This aligns evidence showing that fecal metabolomics reflects gut microbial changes in enteric diseases (Vich Vila et al., 2024).

Similar to butyric acid, histamine (C5H9N3, 111.15 g/mol), a biogenic amine derived from histidine, was significantly elevated in birds affected with NE. The Jejunum, the primary site of infection, exhibited the highest upregulation (LogFC = 3.16), reflecting localized inflammation. Rectal samples showed a notable increase (LogFC = 2.76), suggesting systemic inflammation and microbial dysbiosis. Serum levels, while elevated (LogFC = 0.69), indicated a milder systemic response. The pathway enrichment analysis showed the disturbances in histidine metabolism, highlighting histidine, a precursor metabolite involved in histamine overproduction during NE. These findings highlight histamine’s dysregulation and its impact on immune modulation through histidine metabolism, which is closely associated with bacterial colonization, survival, and virulence in avian intestinal tracts (Krell et al., 2021). Regression analysis showed histamine as a strong predictor of NE-associated disruption in metabolism, demonstrating strong links to metabolites such as N-acetyl-histidine and butyric acid, which are highly relevant in immune modulation and energy metabolism. Correlations of spermine and creatine, metabolites involved in inflammation and epithelial tissue repair, underscoring the contribution of histamine to mucosal integrity and tight junction dynamics, reflecting its multifaceted role in both local and systemic responses to CP infection.

Histamine plays a dual role in intestinal homeostasis, acting as a mediator of both allergic and non-allergic inflammatory processes (Akdis and Blaser, 2003; Li et al., 2015). While beneficial at physiological levels, elevated concentrations are linked to mucosal inflammatory disorders, including irritable bowel syndrome, inflammatory bowel disease, and histamine intolerance, where excessive levels exacerbate epithelial barrier dysfunction and chronic inflammation (Smolinska et al., 2022). The elevated histamine levels observed in this study points out the intricate connection between microbial dysbiosis and CP proliferation, both locally in the jejunum and systemically, as evidenced by its presence in serum and fecal contents. Similar to butyric acid in the gut, histamine can play a protective or negative role against bacterial infections (Metz et al., 2011). A previous study reported significantly higher abundance of histamine-secreting bacteria like CP, *Enterococcus faecalis*, genus *Staphylococcus*, *Proteus*, and unidentified genera of *Enterobacteriaceae* in histamine intolerant patients as compared to healthy individuals (Sánchez-Pérez et al., 2022). Similarly, our findings suggest that CP proliferation and the associated microbiome imbalance potentially induced an increase in histamine levels in the jejunum of NE birds.

The detectability of elevated levels of butyric acid and histamine in serum, feces, and jejunum across both experiments suggests their potential as consistent diagnostic markers for early NE detection. Fecal detection of metabolites is a feasible non-invasive industry technique that does not require any personnel training compared to blood sample collection, hence has the advantage of developing disease diagnostic tool at the farm level. Despite its strengths, this study sincerely acknowledges a few limitations. This includes our study’s-controlled feed design, concurrent infections, and management practices that may partially reflect the actual field conditions. Additionally, a cross-sectional study design restricts us from generalizing and assessing the temporal changes during NE. Although butyric acid and histamine are promising metabolite biomarkers, their specificity and causal roles require further evaluation. For field applications, developing point-of-care metabolite detection devices like portable GC-MS, biosensors, and colorimetric or enzymatic tests for histamine and butyric acid detection might be useful. However, confounding factors such as dietary variations, concurrent infections, and environmental stressors should be considered. For example, dietary fiber or prebiotics can alter gut microbiota and increase beneficial metabolites like butyrate (Shang et al., 2018). Concurrent infections, particularly coccidiosis caused by *Eimeria* spp., disrupt microbiota diversity, potentially influencing metabolite profiles (Jebessa et al., 2022). Environmental stressors, notably heat stress, may independently impact gut barrier integrity and alter metabolites like histamine and butyrate (Shi et al., 2019).

This study concludes that butyric acid and histamine could act as potential metabolite biomarkers for the early, non-invasive diagnosis of NE in broilers. Their increased concentration in serum, feces, and jejunum points out their role in both systemic and local inflammation in NE conditions. Real-time detection of NE in a flock, before the development of any clinical signs or pathological lesions of NE in the intestine, can be developed utilizing metabolomics technology as a rapid diagnostic kit. This approach will benefit poultry producers to improve poultry health and welfare by implementation of timely mitigating strategies. The observed metabolic changes, including disruptions in histidine metabolism and the TCA cycle, underscore the complex interplay between microbial dysbiosis, host metabolism, and inflammation during NE. These biomarkers need further validation in field conditions under different management conditions and must be studied for their application as diagnostic and therapeutic targets by cross-sectional and longitudinal studies in future.

## Data Availability

The original contributions presented in this study are included in this article/Supplementary material, further inquiries can be directed to the corresponding author.

## Footnotes

1
https://github.com/kevinblighe/enhancedvolcano
