# Supplementary material for: Elevated butyric acid and histamine in feces and serum as an indicator of onset of necrotic enteritis in broiler chickens
Source: Front Microbiol. 2025 May 9;16:1581309. doi: 10.3389/fmicb.2025.1581309 (PMC12098369; doi:10.3389/fmicb.2025.1581309)
Supplement: Supplementary file 1 [file Supplementary_file_1.docx]

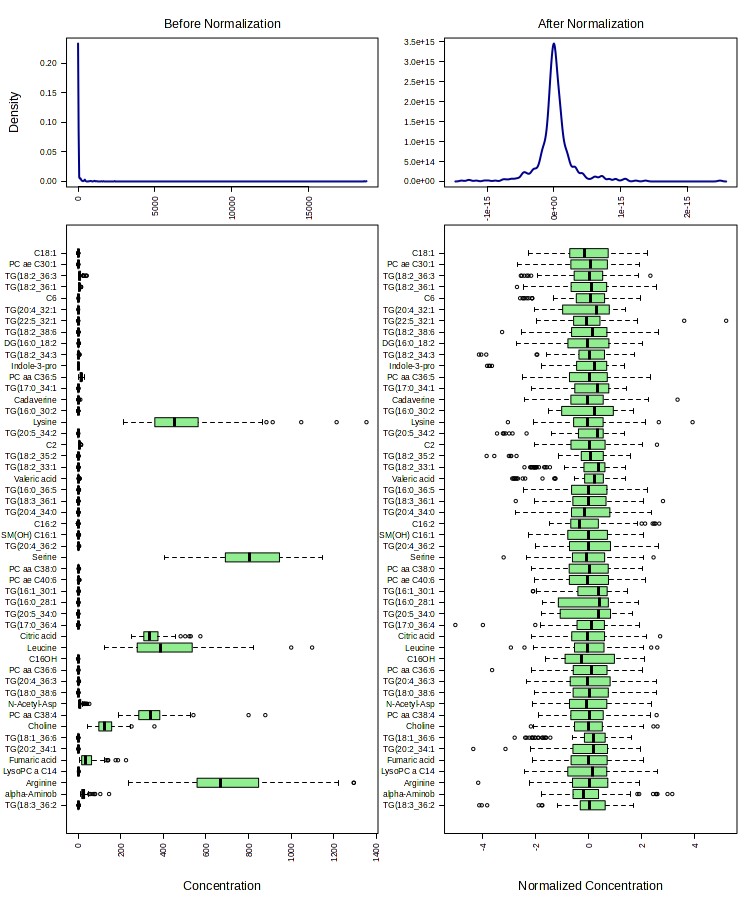


**Supplementary Figure 1**: **Serum metabolomics raw data processing**: Density plots in the left panel highlight shifts in metabolite concentrations due to normalization, while scatter and box plots in the right panel demonstrate clearer distinctions between NE-affected and control samples, enhancing statistical analysis.


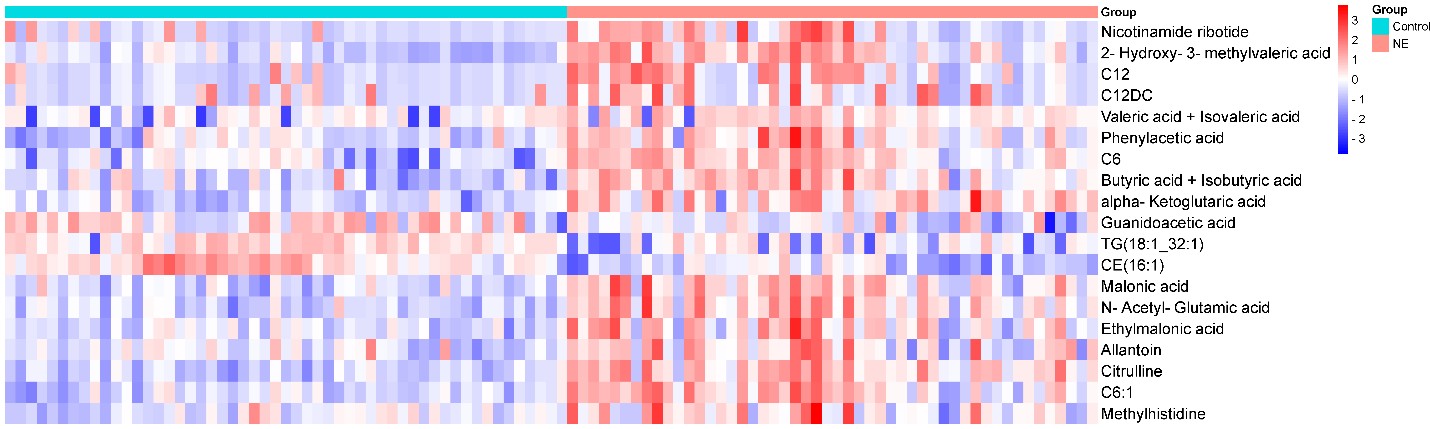


**Supplementary Figure 2: Heatmap of metabolites from Serum samples in NE Birds:** Heatmap showing the top 20 differentially expressed metabolites between NE and control (CN) serum samples, with red indicating higher expression and blue indicating lower expression.


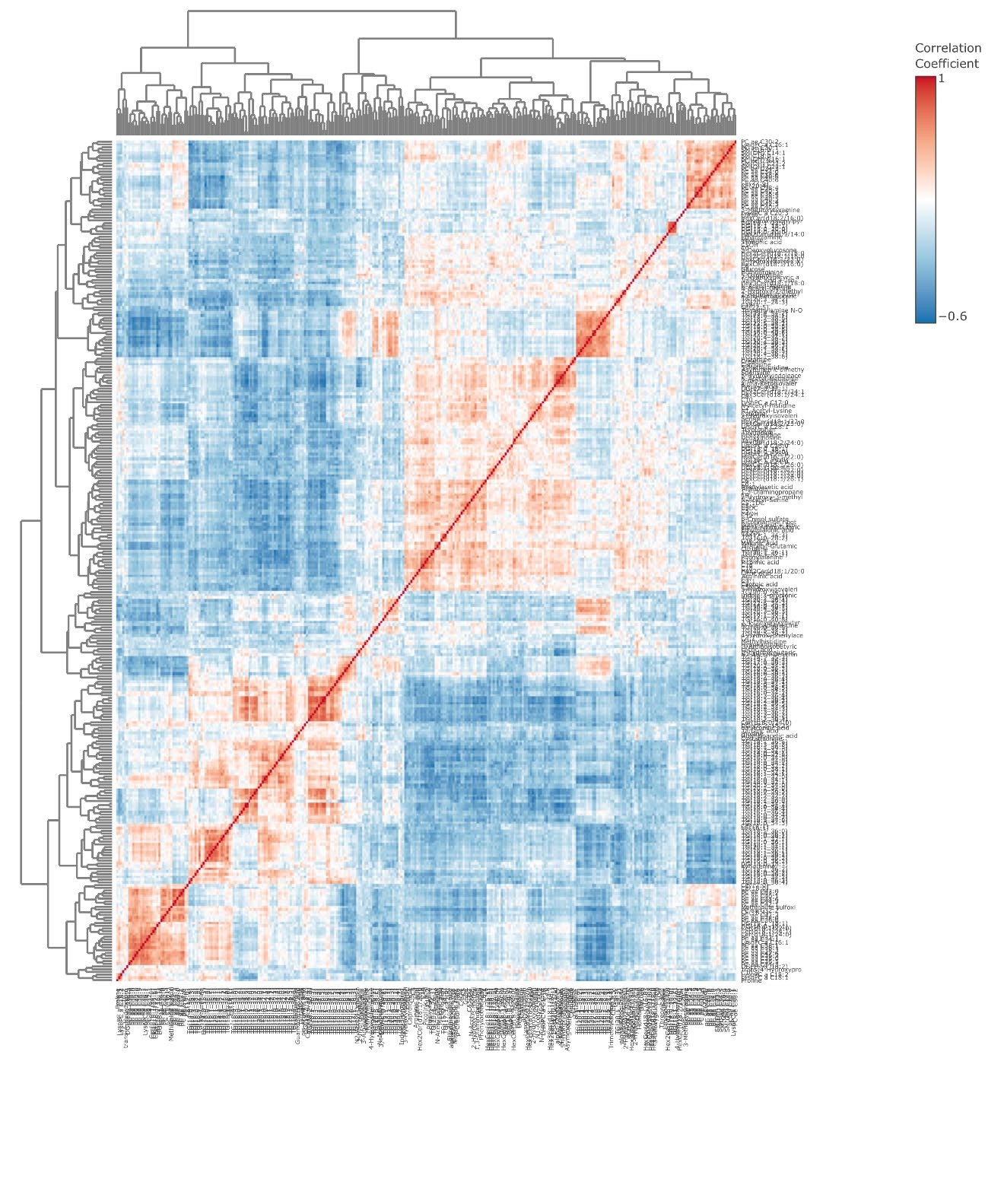


**Supplementary Figure 3: Correlation analysis plots of metabolites from Serum samples in NE Birds:** A. Correlation Plot showing Pairwise correlations among key metabolites, with red showing positive and blue showing negative correlations. Darker shades indicate stronger relationships.


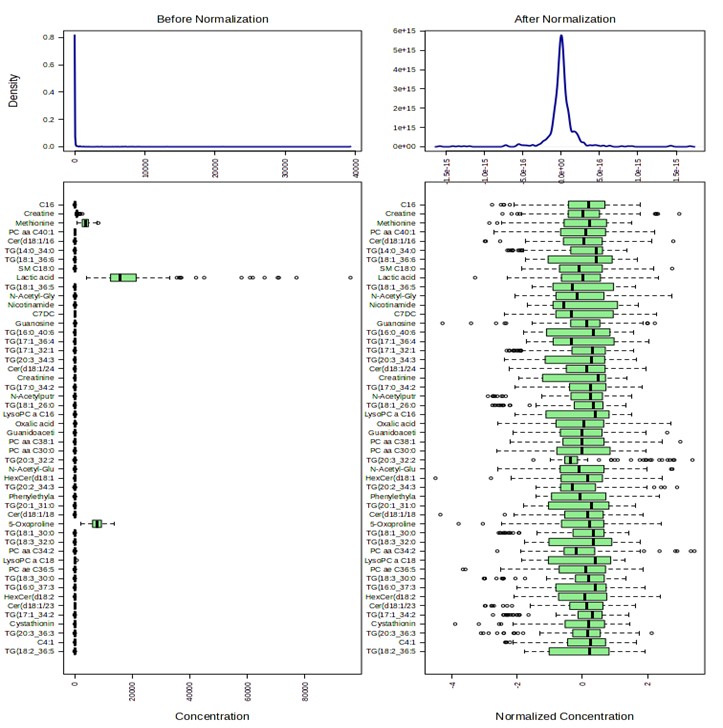


**Supplementary Figure 4: Jejunal metabolomics raw data processing**: Density plots in the left panel highlight shifts in metabolite concentrations due to normalization, while scatter and box plots in the right panel demonstrate clearer distinctions between NE affected and control samples, enhancing statistical analysis.


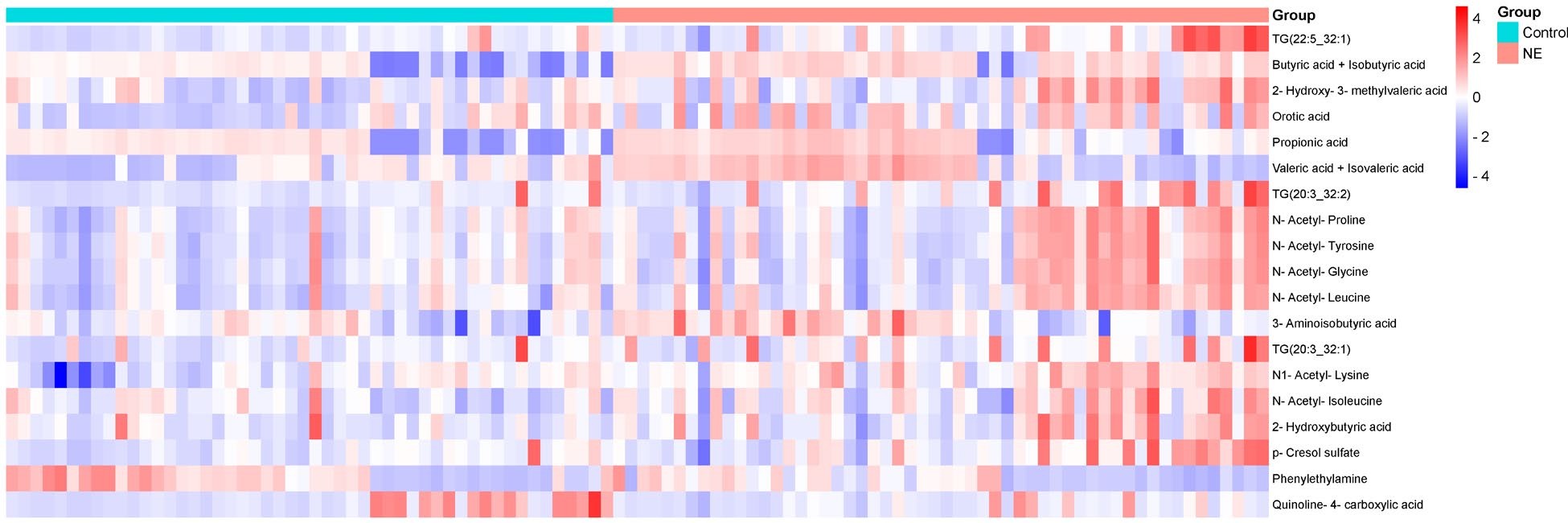


**Supplementary Figure 5: Heatmap analysis of Jejunal Samples in NE Birds:** (B) Heatmap displaying the top 20 differentially expressed metabolites between NE-affected and control jejunal samples. The color gradient indicates the relative expression levels of metabolites:


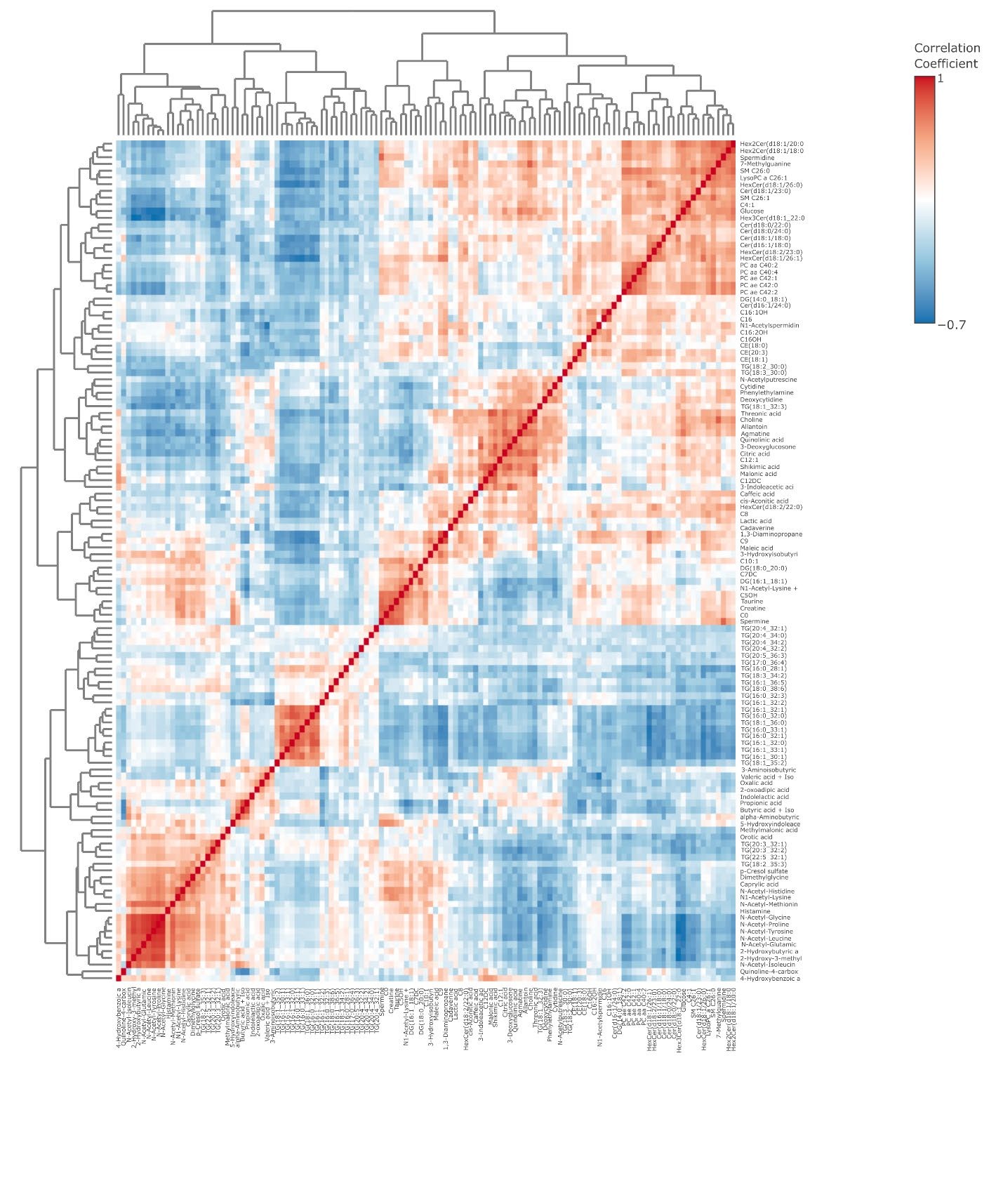


**Supplementary Figure 6: Metabolite correlation analysis of Jejunal samples in NE Birds:** Correlation Plot showing Pairwise correlations among key metabolites, with red showing positive and blue showing negative correlations. Darker shades indicate stronger relationships.


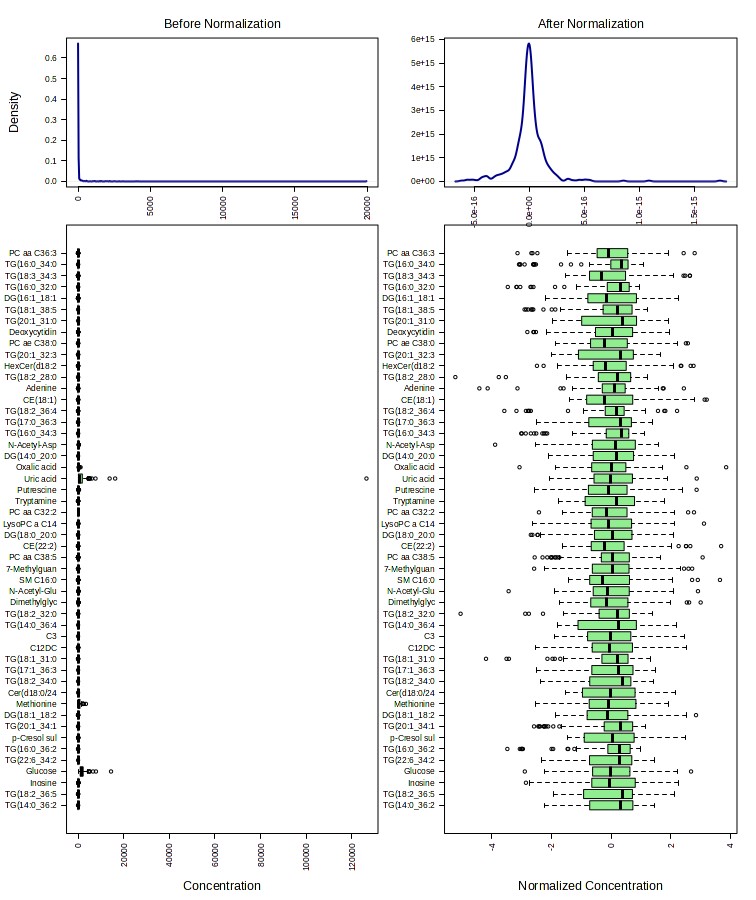


**Supplementary Figure 7: Fecal samples metabolomics raw data processing**: Density plots in the left panel highlight shifts in metabolite concentrations due to normalization, while scatter and box plots in the right panel demonstrate clearer distinctions between NE affected and control samples, enhancing statistical analysis.


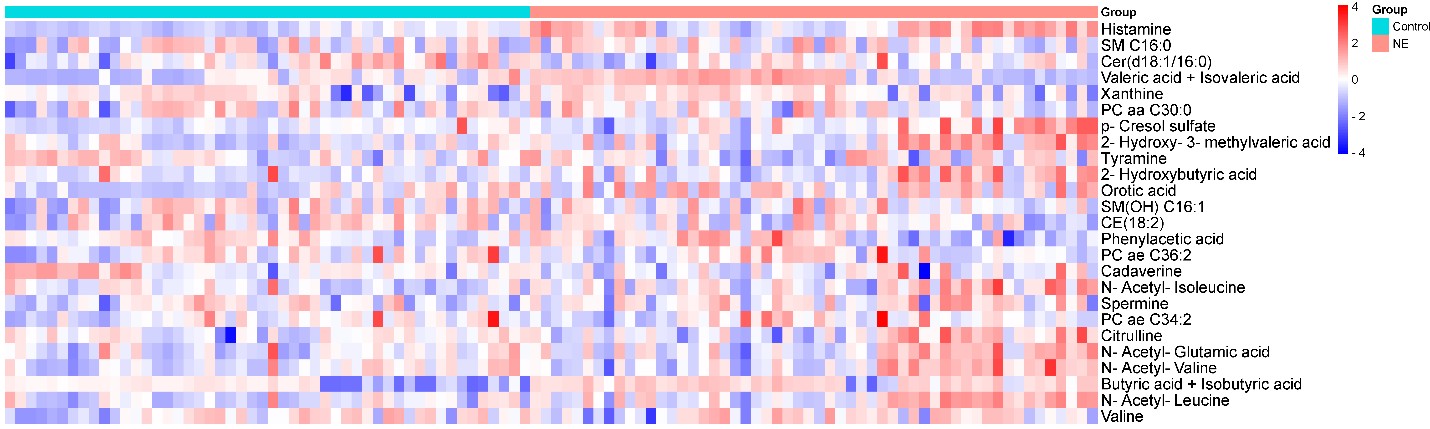


**Supplementary Figure 8**: **Heatmap analysis of rectal Samples in NE Birds:** Heatmap displaying the top 20 differentially expressed metabolites between NE-affected and control jejunal samples. The color gradient indicates the relative expression levels of metabolites:


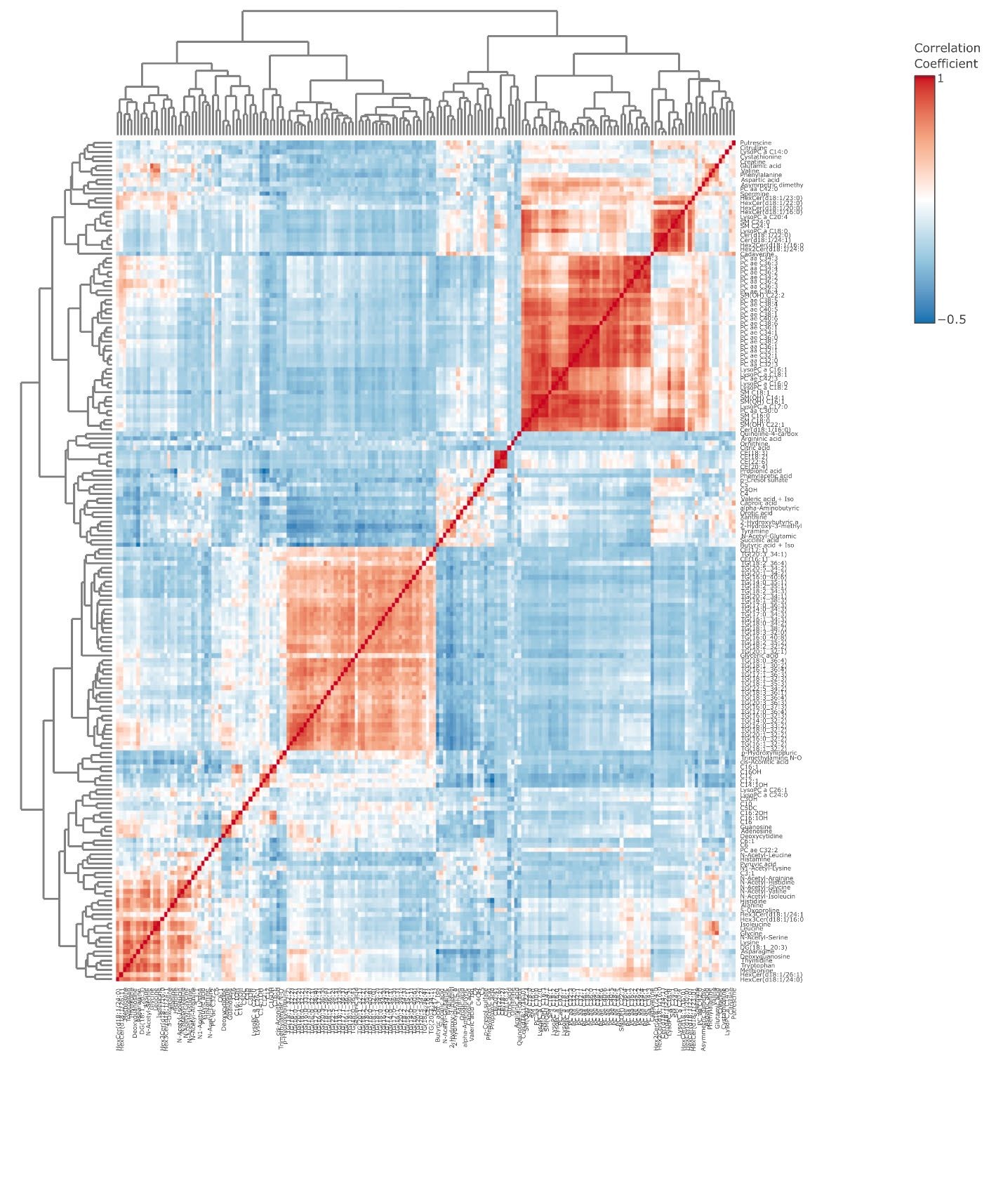


**Supplementary Figure 9: Metabolite correlation analysis of rectal samples in NE Birds:** Correlation Plot showing Pairwise correlations among key metabolites, with red showing positive and blue showing negative correlations. Darker shades indicate stronger relationships.


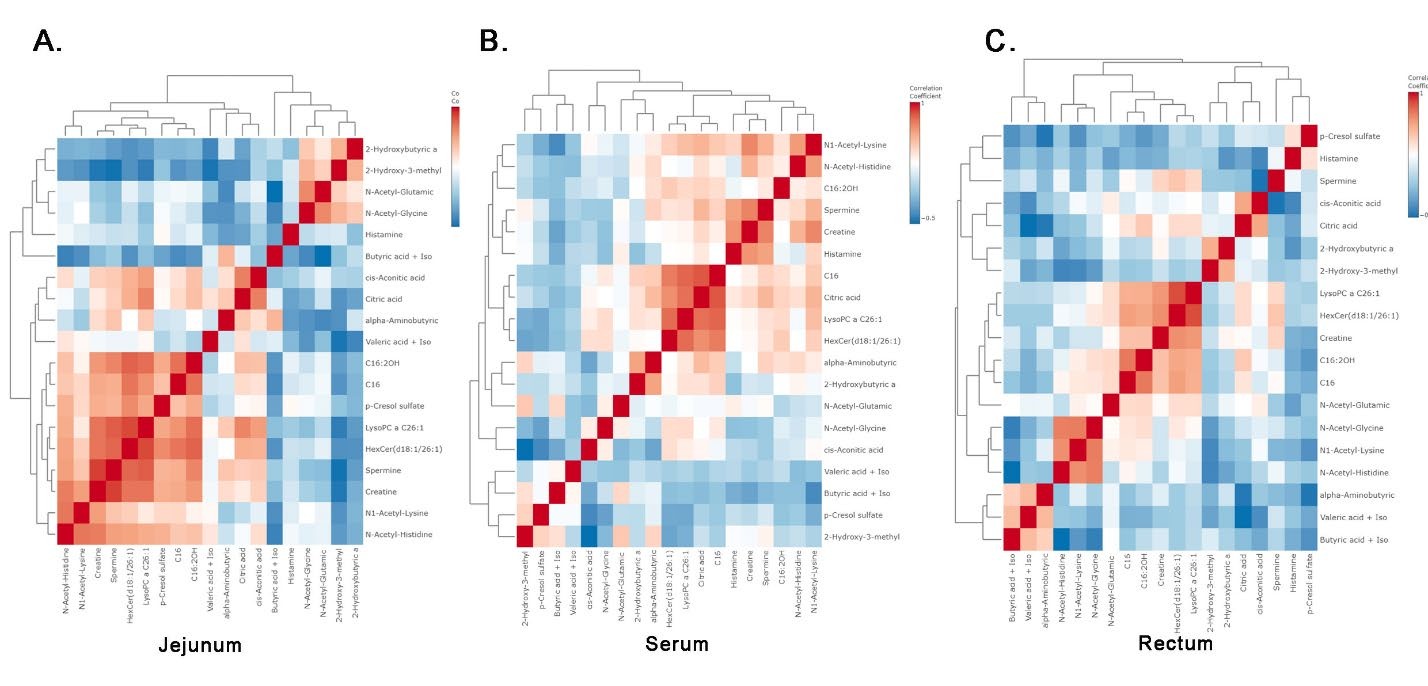


**Supplementary Figure 10: Metabolite correlation analysis of Common Metabolites Across Serum, Jejunal contents and Feces in NE Birds:** Correlation Plot showing Pairwise correlations among key metabolites across jejunum (A), serum (S), and fecal contents (C), with red showing positive and blue showing negative correlations. Darker shades indicate stronger relationships.


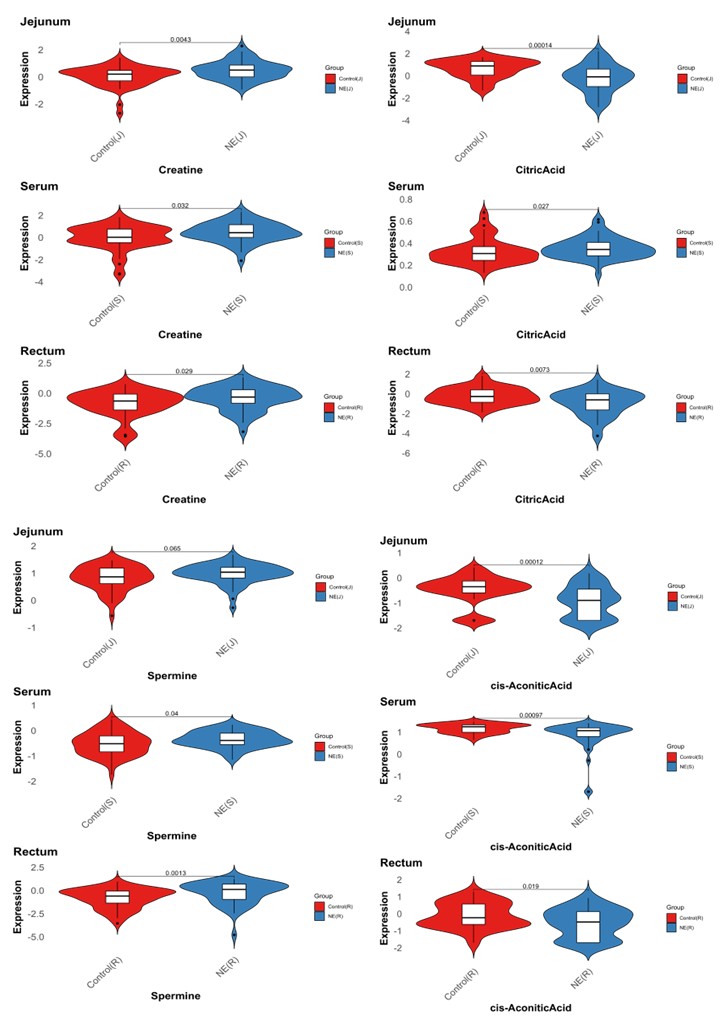


**Supplementary Figure 11: Expression status of Creatinine, Citric acid, Spermine and cis-Aconitic acid metabolites across serum, fecal and jejunal samples in NE birds**. Violin plots illustrate the distribution and central tendency differences in fold changes (log2 scale) of histamine and butyric acid across serum, fecal, and jejunal samples between NE-affected and control groups (*p* < 0.05). Positive values on the y-axis indicate upregulation, while negative values represent downregulation.
